# Drug-resistant Diarrheogenic *Escherichia coli*, Mexico

**DOI:** 10.3201/eid1108.050192

**Published:** 2005-08

**Authors:** Teresa Estrada-García, Jorge F. Cerna, Leova Paheco-Gil, Raúl F. Velázquez, Theresa J. Ochoa, Javier Torres, Herbert L. DuPont

**Affiliations:** * Centro de Investigación y de Estudios Avanzados del Instituto Politécnico Nacional, Mexico City, Distrito Federal, Mexico;; † Instituto Mexicano del Seguro Social, Mexico City, Distrito Federal, Mexico;; ‡ Universidad Juárez Autónoma de Tabasco, Villahermosa, Tabasco, Mexico;; § Hospital del Niño “Dr. Rodolfo Nieto Padrón,” Villahermosa, Tabasco, Mexico;; ¶ University of Texas Health Science Center at Houston, Houston, Texas, USA;; # Baylor Collage of Medicine, Houston, Texas, USA;; ** St. Luke's Episcopal Hospital, Houston, Texas, USA

**Keywords:** Pediatric Infectious Diseases/vaccines, AOM, bacterial respiratory infection, S. pneumoniae, antibiotic treatment, antibiotic tr, MOLECULAR EPIDEMIOLOGY, gastroenteritis

## Abstract

Diarrheogenic *Escherichia coli* isolates from 45 (73%) of 62 hospitalized patients were resistant to common antimicrobial drugs. Sixty-two percent were multidrug resistant, and >70% were resistant to trimethoprim-sulfamethoxazole and ampicillin. Ciprofloxacin and cefotaxime were uniformly active. Effective and safe oral agents are needed to treat children with bacterial diarrhea.

The best characterized diarrheogenic *Escherichia coli* (DE) groups include enterotoxigenic (ETEC), enteropathogenic (EPEC), enteroinvasive (EIEC), enteroaggregative (EAEC), and Shiga toxin–producing (STEC) *E. coli*. STEC is also known as verotoxin-producing or enterohemorrhagic *E. coli*. Except for slow-fermenting sorbitol STEC (as O157:H7), DE are not routinely sought as stool pathogens in clinical laboratories worldwide, perhaps because rapid and sensitive laboratory techniques are lacking. However, DE are a leading cause of children's diarrhea in developing countries (1), and some are increasingly being recognized as important enteropathogens in developed countries (1,2). To establish the prevalence and resistance patterns of these microorganisms in hospitalized children in Mexico, we conducted a prospective study. *E. coli* strains were analyzed by using 2 comprehensive multiplex polymerase chain reaction (PCR) assays, and strains harboring DE genes were analyzed for their antimicrobial resistance patterns.

## The Study

Two groups of children <5 years of age hospitalized for acute diarrhea were studied: 1) 285 children enrolled from March 2000 to February 2001 at 3 main hospitals of Mexico City, Instituto Mexicano del Seguro Social (IMSS); and 2) 145 children enrolled from February to October 2004 at the Children's Hospital in Villahermosa, Tabasco, Hospital del Niño, Secretaría de Salud (SS). The institutional review boards of IMSS and SS approved these studies, and parental informed consent was obtained for each patient. Children were included if they had ≥3 loose stools in 24 hours or an episode of bloody diarrhea. Children were excluded if they had received previous antimicrobial drug treatment. A total of 430 Mexican children hospitalized for acute diarrhea (<14 days) were included, 222 (52%) were male and 321 (77%) were ≤2 years of age. Stool diagnostic evaluations were done by standard laboratory procedures, including culture for *Salmonella* spp., *Shigella* spp., *Vibrio cholerae*, and *Campylobacter* spp.; enzyme-linked immunosorbent assay or latex agglutination test for rotavirus; and microscopy for *Entamoeba histolytica*, *Cryptosporidium parvum*, *Cyclospora cayetanensis*, *Isospora* spp., and *Giardia lamblia*. In addition, from all 430 stool samples, 5 lactose-fermenting colonies and 5 sorbitol-nonfermenting colonies with shapes resembling that of *E. coli* were selected from standard and sorbitol MacConkey agar plates, respectively and speciated biochemically. A total of 2,010 *E. coli* strains from 430 patients were selected, and all were analyzed by a multiplex PCR (3) that detects the following pathogenic genes: heat-stable and heat-labile enterotoxins (*st*, *lt*) for ETEC, intimin (*eae*A) and bundle-forming pilus (*bfp*) for EPEC, Shiga toxin 1 and 2 (*stx*1, *stx*2) and intimin (*eae*A) for STEC, and invasion-associated loci (*ia*l) for EIEC. STEC from patients were further characterized by the expression of the O157 lipopolysaccharide antigen and enterohemolysin gene (*hly*A) by using latex particle agglutination kit (Oxoid Limited, Basingstoke, UK) and PCR, respectively. Moreover, all *E. coli* strains from the Villahermosa study were analyzed by a second multiplex PCR that detects 3 plasmidborne virulence genes (*aap*, *agg*R, and *aat*A) from EAEC (4). Both PCRs were developed at the Department of Molecular Biomedicine, Centro de Investigación y de Estudios Avanzados (CINVESTAV). In the present study, pathogenic EAEC were defined as those harboring the 3 plasmidborne genes, *aap*, *agg*R, and *aat*A, as previously described (4). This definition may be stringent, but for the antimicrobial susceptibility analysis, we wanted to include only pathogenic DE when possible. Finally, *E. coli* strains positive for any DE gene were analyzed for their antimicrobial susceptibility by disk diffusion, according to the Clinical and Laboratory Standards Institute (formerly NCCLS) guidelines (5).

DE were identified in 62 (14%) of 430 patients, the second highest proportion after that of rotavirus (41%). Other pathogens isolated were *Shigella* spp. (9%), *Salmonella* spp. (3%), *Cryptosporidium* spp. (0.9%), and *Campylobacter* spp. (0.7%). Of the 2,120 analyzed strains, 170 (8%) were positive for at least 1 DE gene. As shown in Table 1, ETEC was the most prevalent DE group isolated in patients, closely followed by EAEC (only characterized in the Villahermosa study) and then by atypical EPEC (aEPEC [*eae*A+ *bfp*–]). Most STEC strains were *stx*2+ or *stx*1+ *eae*A+, none expressed the O157 lipopolysaccharide antigen, and only 1 contained the *hly*A gene.

**Table 1:** Diarrheogenic Escherichia coli (DE) isolated from patients with diarrhea

DE group*	No. patients by DE group (%)	No. strains† by DE group	Strain genotypes and no. positive strains by gene(s)
ETEC	17 (27)	50	*lt* = 29, *st* = 17, *st*-*lt* = 4
EAEC	16 (26)	56	*aap*-*agg*R-*aat*A = 56
aEPEC	13 (21)	31	*eae*A = 31
STEC	11 (18)	22	*stx*2 = 9, *stx*1-*eae*A = 9, *stx*1 = 3, *stx*1-*eae*A-*hly*A = 1
EPEC	3 (5)	7	*eae*A-*bfp* = 7
EIEC	2 (3)	4	*ia*l = 4
Total	62	170	

*ETEC, enterotoxigenic *E. coli*; EAEC, enteroaggregative *E. coli*; aEPEC, atypical enteropathogenic *E. coli*; STEC, Shiga toxin–producing *E. coli*; EPEC, enteropathogenic *E. coli*; EIEC, enteroinvasive *E. coli*. †All diarrheogenic *E. coli* strains isolated from patients with diarrhea; in general, 5 *E. coli* strains were isolated from each patient.

Antimicrobial resistance patterns for patients that had DE included 65% resistant to trimethoprim-sulfamethoxazole (TMP-SMX) and 73% to ampicillin (Table 2). Resistance to ≥3 antimicrobial drugs (multidrug resistance) was 58%. The antimicrobial resistance patterns for the strains were similar to the ones described above for the patients. Thus, of the 170 strains 105 (62%) were multidrug resistant, 145 (85%) were resistant to tetracycline, 124 (73%) to ampicillin, 127 (75%) to TMP-SMX, 29 (17%) to chloramphenicol, 4 (2%) to gentamicin, and none to ciprofloxacin and cefotaxime. Most isolated strains per patient showed similar susceptibility. Comparison of resistance patterns between patients belonging to different DE groups showed that aEPEC was significantly less resistant (p<0.05, chi-square test) to ampicillin and TMP-SMX than ETEC and EAEC.

**Table 2:** Diarrheogenic Escherichia coli resistance patterns*†

No. patients	Tet, n (%)	Amp, n (%)	TMP-SMX, n (%)	Chlor, n (%)	MDR, n (%)
ETEC (n = 17)	16 (94)	15 (88)	12 (71)	2 (12)	11 (65)
EAEC (n = 16)	15 (94)	13 (81)	14 (88)	3 (19)	11 (69)
aEPEC (n = 13)	6 (46)	5 (38)	5 (38)	2 (15)	5 (38)
STEC (n = 11)	9 (81)	8 (72)	7 (63)	4 (36)	7 (63)
EPEC (n = 3)	3 (100)	3 (100)	2 (67)	0	2 (67)
EIEC (n = 2)	1 (50)	1 (50)	0	1 (50)	1 (50)
Total (n = 62)	51 (82)	45 (73)	40 (65)	12 (19)	36 (58)

*All isolates were susceptible to ciprofloxacin and cefotaxime. One STEC (9% of STEC or 2% of all isolates) was resistant to gentamicin; all other isolates were susceptible. †Tet, tetracycline; Amp, ampicillin; TMP-SMX, trimethoprim-sulfamethoxazole; Chlor, chloramphenicol; MDR, multidrug resistant; ETEC, enterotoxigenic *E. coli*; EAEC, enteroaggregative *E. coli*; aEPEC, atypical enteropathogenic *E. coli*; STEC, Shiga toxin–producing *E. coli*; EPEC, enteropathogenic *E. coli*; EIEC, enteroinvasive *E. coli*.

## Conclusions

Diarrheogenic *E. coli* prototypes cause high rates of persistent diarrhea (6–8), which has been associated with malnutrition, growth impairment, and death, in developing countries (1,6,8). From the 62 patients with DE, ETEC was the most prevalent group (27% of cases), showing that ETEC continues to be a major health problem in developing countries (6). EAEC accounted for 26% of cases, although it was characterized at only 1 site, and a stringent definition of pathogenic EAEC was used. EAEC prevalence may be even higher, and it may be responsible for much acute diarrhea requiring hospitalization in children, as recently shown in the United States (2). Unexpectedly, 21% of patients harbored aEPEC strains; the pathogenic role of this emerging *E. coli* group is still unclear, but it has been associated with acute (9) and persistent diarrhea (10). In addition, those aEPEC strains showed significantly less resistance (p<0.05, chi-square test) to ampicillin and TMP-SMX than ETEC and EAEC strains, which suggests that aEPEC strains may have recently been acquired in Mexico. Finally, 18% of patients harbored STEC non-O157 strains, showing its role in acute diarrhea that requires hospitalization in Mexico. Together these observations highlight the role of DE in children's diarrhea that requires hospitalization and stress the importance of seeking DE in children's stools by using multiplex PCR technology. We have also shown that specific and sensitive multiplex PCR technology (3,4) that recognizes a diversity of loci in *E. coli* may be cost effective, which would allow clinical laboratories worldwide to identify these pathogens.

Since some DE infections appear indistinguishable from viral gastroenteritis, isolation and identification of DE strains could allow caretakers to provide appropriate treatment for pathogen-specific illness. Oral rehydration therapy (ORT) in children with dehydrating forms of diarrhea has reduced death rates worldwide. ORT, however, does not shorten duration of illness and shedding, whereas antimicrobial therapy may be of value for some forms of DE diarrhea (11). Antimicrobial therapy may be indicated in children with DE diarrhea that is promptly identified and in children with persistent diarrhea. We have shown that most DE strains that cause diarrhea in hospitalized children in Mexico are resistant to TMP-SMX and ampicillin, drugs commonly used to treat pediatric diarrhea. This resistance pattern is an emerging problem for DE strains isolated from children in other developing countries (12) and for other enterobacteria worldwide (12–14). All strains were sensitive to ciprofloxacin and cefotaxime; however, ciprofloxacin and other quinolones are not approved for children because of the risk of damage to immature joints (14), and most parenteral third-generation cephalosporins (e.g., cefotaxime) are administered only in a hospital setting. These results show the need for new, affordable, and safe oral antimicrobial drugs to treat enterobacterial infections in children.
